# Supplementary material for: Rapid Classification of Clostridioides difficile Strains Using MALDI-TOF MS Peak-Based Assay in Comparison with PCR-Ribotyping
Source: Microorganisms. 2021 Mar 23;9(3):661. doi: 10.3390/microorganisms9030661 (PMC8004610; doi:10.3390/microorganisms9030661)
Supplement: Supplementary file 1 [file microorganisms-09-00661-s001.zip › Supplementary materials/Supplementary Table 1.docx]

| **Mass (Da)** | **PTTA** | **PWKW** | **Ave ± SD PR1** | **Ave ± SD PR2** | **Ave ± SD PR3** | **Ave ± SD PR4** | **Ave ± SD PR5** | **Ave ± SD PR6** | **Ave ± SD PR7** | **Ave ± SD PR8** | **Ave ± SD PR9** | **Ave ± SD PR10** |
| --- | --- | --- | --- | --- | --- | --- | --- | --- | --- | --- | --- | --- |
| 2232 | < 0.000001 | < 0.000001 | 7 ± 3 | 7 ± 2 | 9 ± 5 | 6 ± 2 | 10 ± 5 | 14 ± 3 | 9 ± 3 | 6 ± 2 | 11 ± 5 | 57 ± 27 |
| 2641 | < 0.000001 | < 0.000001 | 91 ± 52 | 81 ± 53 | 52 ± 39 | 34 ± 21 | 101 ± 32 | 49 ± 11 | 22 ± 6 | 12 ± 4 | 95 ± 12 | 55 ± 10 |
| 2665 | < 0.000001 | < 0.000001 | 5 ± 2 | 12 ± 9 | 5 ± 3 | 4 ± 2 | 7 ± 2 | 4 ± 1 | 4 ± 1 | 3 ± 1 | 6 ± 2 | 3 ± 1 |
| 3107 | < 0.000001 | < 0.000001 | 20 ± 12 | 19 ± 14 | 18 ± 14 | 12 ± 7 | 26 ± 7 | 196 ± 11 | 8 ± 1 | 8 ± 2 | 28 ± 9 | 17 ± 5 |
| 3220 | < 0.000001 | < 0.000001 | 78 ± 18 | 52 ± 21 | 102 ± 69 | 65 ± 34 | 69 ± 41 | 22 ± 42 | 71 ± 10 | 50 ± 9 | 41 ± 7 | 25 ± 5 |
| 3661 | < 0.000001 | < 0.000001 | 18 ± 9 | 16 ± 18 | 24 ± 39 | 14 ± 20 | 45 ± 9 | 12 ± 5 | 5 ± 1 | 4 ± 1 | 49 ± 9 | 46 ± 9 |
| 4297 | < 0.000001 | < 0.000001 | 21 ± 17 | 28 ± 15 | 43 ± 20 | 32 ± 16 | 17 ± 8 | 44 ± 3 | 36 ± 6 | 44 ± 7 | 11 ± 3 | 15 ± 3 |
| 4426 | < 0.000001 | < 0.000001 | 33 ± 10 | 36 ± 10 | 48 ± 36 | 40 ± 8 | 33 ± 17 | 47 ± 13 | 46 ± 5 | 41 ± 3 | 18 ± 4 | 505 ± 219 |
| 4657 | < 0.000001 | < 0.000001 | 35 ± 10 | 42 ± 32 | 43 ± 10 | 44 ± 11 | 41 ± 25 | 102 ± 6 | 56 ± 5 | 56 ± 5 | 30 ± 10 | 43 ± 9 |
| 5020 | < 0.000001 | < 0.000001 | 56 ± 22 | 50 ± 24 | 49 ± 28 | 52 ± 35 | 65 ± 26 | 10 ± 19 | 36 ± 8 | 62 ± 10 | 88 ± 12 | 61 ± 12 |
| 5314 | < 0.000001 | < 0.000001 | 14 ± 5 | 17 ± 11 | 14 ± 5 | 18 ± 9 | 13 ± 4 | 8 ± 2 | 16 ± 2 | 14 ± 3 | 15 ± 4 | 22 ± 7 |
| 5346 | < 0.000001 | < 0.000001 | 16 ± 5 | 20 ± 12 | 16 ± 8 | 22 ± 10 | 21 ± 9 | 459 ± 2 | 19 ± 3 | 14 ± 2 | 55 ± 6 | 24 ± 7 |
| 5760 | < 0.000001 | < 0.000001 | 150 ± 163 | 99 ± 63 | 116 ± 154 | 177 ± 156 | 98 ± 41 | 42 ± 170 | 106 ± 25 | 137 ± 19 | 113 ± 43 | 258 ± 48 |
| 5961 | < 0.000001 | < 0.000001 | 87± 60 | 109 ± 64 | 140 ± 75 | 128 ± 77 | 43 ± 29 | 20 ± 11 | 161± 17 | 197 ± 20 | 16 ± 6 | 19 ± 3 |
| 6396 | < 0.000001 | < 0.000001 | 25 ± 19 | 33 ± 22 | 43 ± 20 | 45 ± 25 | 32 ± 17 | 61 ± 11 | 58 ± 6 | 48 ± 5 | 39 ± 21 | 7 ± 1 |
| 6892 | < 0.000001 | < 0.000001 | 62 ± 42 | 97 ± 59 | 120 ± 75 | 75 ± 42 | 29 ± 16 | 39 ± 17 | 131 ± 24 | 162 ± 36 | 18 ± 3 | 15 ± 3 |
| 7093 | < 0.000001 | < 0.000001 | 58 ± 40 | 63 ± 44 | 103 ± 53 | 184 ± 86 | 54 ± 46 | 49 ± 33 | 106 ± 11 | 248 ± 51 | 20 ± 10 | 65 ± 35 |

**Supplementary Table 1. Discriminating peaks obtained for the first classifying algorithm model based on 10 ribotypes.**

Legend. Da: Dalton; PTTA: *p*-value obtained by ANOVA test; PWKW: *p*-value obtained by Kruskal-Wallis test; Ave: the peak area/intensity average; SD: standard deviation.
