# Supplementary material for: Rapid Classification of Clostridioides difficile Strains Using MALDI-TOF MS Peak-Based Assay in Comparison with PCR-Ribotyping
Source: Microorganisms. 2021 Mar 23;9(3):661. doi: 10.3390/microorganisms9030661 (PMC8004610; doi:10.3390/microorganisms9030661)
Supplement: Supplementary file 1 [file microorganisms-09-00661-s001.zip › Supplementary materials/Supplementary Table 2.docx]

**Supplementary Table 2. Discriminating peaks obtained for the second classifying algorithm model based on the 5 most frequent ribotypes.**

| **Mass (Da)** | **PTTA** | **PWKW** | **Ave ± SD PR1** | **Ave ± SD PR2** | **Ave ± SD PR3** | **Ave ± SD PR4** | **Ave ± SD PR5** |
| --- | --- | --- | --- | --- | --- | --- | --- |
| 2616 | < 0.000001 | < 0.000001 | 59 ± 31 | 59 ± 33 | 67 ± 15 | 43 ± 20 | 88 ± 28 |
| 2641 | < 0.000001 | < 0.000001 | 111 ± 59 | 98 ± 55 | 114 ± 28 | 39 ± 25 | 134 ± 43 |
| 2675 | < 0.000001 | < 0.000001 | 22 ± 8 | 200 ± 250 | 27 ± 10 | 18 ± 8 | 36 ± 14 |
| 3220 | < 0.000001 | < 0.000001 | 88 ± 50 | 55 ± 22 | 75 ± 24 | 65 ± 34 | 64 ± 39 |
| 3446 | < 0.000001 | < 0.000001 | 82 ± 39 | 107 ± 57 | 93 ± 12 | 63 ± 27 | 45 ± 20 |
| 3699 | < 0.000001 | < 0.000001 | 147 ± 107 | 112 ± 95 | 132 ± 44 | 70 ± 62 | 215 ± 129 |
| 3963 | < 0.000001 | < 0.000001 | 927 ± 602 | 647 ± 611 | 804 ± 309 | 207 ± 271 | 1023 ± 626 |
| 4983 | < 0.000001 | < 0.000001 | 224 ± 68 | 212 ± 64 | 138 ± 39 | 180 ± 86 | 130 ± 56 |
| 5034 | < 0.000001 | 0.00000257 | 63 ± 26 | 62 ± 27 | 50 ± 9 | 80 ± 33 | 59 ± 16 |
| 5438 | < 0.000001 | < 0.000001 | 207 ± 161 | 239 ± 181 | 101 ± 59 | 238 ± 150 | 64 ± 48 |
| 5760 | 0.0000247 | < 0.000001 | 99 ± 116 | 98 ± 63 | 122 ± 89 | 191 ± 157 | 82 ± 41 |
| 6368 | < 0.000001 | < 0.000001 | 145 ± 105 | 184 ± 127 | 114 ± 22 | 249 ± 159 | 59 ± 44 |
| 7078 | < 0.000001 | < 0.000001 | 140 ± 72 | 158 ± 106 | 106 ± 23 | 100 ± 45 | 56 ± 54 |
| 7093 | < 0.000001 | < 0.000001 | 57 ± 43 | 60 ± 42 | 60 ± 31 | 165 ± 76 | 43 ± 39 |
| 7265 | < 0.000001 | < 0.000001 | 321 ± 231 | 208 ± 195 | 148 ± 83 | 84 ± 119 | 252 ± 200 |
| 7396 | < 0.000001 | < 0.000001 | 141 ± 137 | 74 ± 77 | 84 ± 57 | 32 ± 52 | 141 ± 105 |
| 9655 | < 0.000001 | < 0.000001 | 121 ± 91 | 163± 122 | 90 ± 27 | 262 ± 98 | 66 ± 67 |

Legend. Da: Dalton; PTTA: *p*-value obtained by ANOVA test; PWKW: *p*-value obtained by Kruskal-Wallis test; Ave: the peak area/intensity average; SD: standard deviation.
